# Sequential dual-drug delivery of BMP-2 and alendronate from hydroxyapatite-collagen scaffolds for enhanced bone regeneration

**DOI:** 10.1038/s41598-020-80608-3

**Published:** 2021-01-12

**Authors:** Dongtak Lee, Maierdanjiang Wufuer, Insu Kim, Tae Hyun Choi, Byung Jun Kim, Hyo Gi Jung, Byoungjun Jeon, Gyudo Lee, Ok Hee Jeon, Hak Chang, Dae Sung Yoon

**Affiliations:** 1 School of Biomedical Engineering, Korea University, Seoul, 02841 Republic of Korea; 2 Department of Plastic and Reconstructive Surgery, College of Medicine, Seoul National University, Seoul, 03080 Republic of Korea; 3 Interdisciplinary Program in Bioengineering, Graduate School, Seoul National University, Seoul, 03080 Republic of Korea; 4 Department of Biotechnology and Bioinformatics, Korea University, Sejong, 30019 Republic of Korea; 5 Department of Biomedical Sciences, College of Medicine, Korea University, Seoul, 02841 Republic of Korea; 6 Interdisciplinary Program in Precision Public Health, Korea University, Seoul, 02841 Republic of Korea

**Keywords:** Trauma, Preclinical research, Biomedical engineering, Biomedical materials, Drug delivery

## Abstract

The clinical use of bioactive molecules in bone regeneration has been known to have side effects, which result from uncontrolled and supraphysiological doses. In this study, we demonstrated the synergistic effect of two bioactive molecules, bone morphogenic protein-2 (BMP-2) and alendronate (ALN), by releasing them in a sequential manner. Collagen-hydroxyapatite composite scaffolds functionalized using BMP-2 are loaded with biodegradable microspheres where ALN is encapsulated. The results indicate an initial release of BMP-2 for a few days, followed by the sequential release of ALN after two weeks. The composite scaffolds significantly increase osteogenic activity owing to the synergistic effect of BMP-2 and ALN. Enhanced bone regeneration was identified at eight weeks post-implantation in the rat 8-mm critical-sized defect. Our findings suggest that the sequential delivery of BMP-2 and ALN from the scaffolds results in a synergistic effect on bone regeneration, which is unprecedented. Therefore, such a system exhibits potential for the application of cell-free tissue engineering.

## Introduction

The successful restoration of large bone defects caused by trauma, tumor ablation, or metabolic diseases remains a significant clinical challenge in reconstructive surgery. Autogenous bone grafts are limited by their source, and allografts face immune rejection^1,2^. Bone tissue engineering has attracted considerable attention as a promising alternative strategy for reconstructing bone defects and inducing tissue regeneration^3,4^. Conventional tissue engineering involves using a biomaterial with bioactive molecules and cells. This combination may suffer from problems such as long-term cell culture for new bone formation, including the expense of cell transplantation and preservation as well as the risk of contamination and cell rejection^5^. Alternatively, in situ bone tissue engineering by mimicking a natural microenvironment with cell-free, osteoinductive biomaterials can provide a better solution. It is directly implanted in the graft sites with biomolecules for recruiting host circulating stem cells in situ, such as bone mesenchymal stem cells (BMSCs) for bone regeneration^6^.

Biomaterials are crucial elements of in situ bone tissue engineering because they provide a three-dimensional (3D) scaffold that facilitates the formation of the extracellular matrix (ECM) and supports cell growth^7,8^. Collagen, which is a biologically derived protein, has been approved as an efficient bone tissue engineering material because it effectively preserves bioactive molecule activity and can assist the osteogenic differentiation of BMSCs^9^. Moreover, nano-hydroxyapatite particles (nHAp) exhibit excellent osteoconductivity because of chemical similarity to natural bone^10,11^. The incorporation of nHAp in collagen scaffolds promotes bone regeneration through the osteoinductive effects of nHAp on BMSCs, as well as increasing the mechanical properties of collagen scaffolds^12,13^.

Bioactive molecules are crucial elements in bone tissue engineering for enhancing the functionality of biomaterials to promote the recruitment and osteogenic differentiation of BMSCs^7^. Recombinant human bone morphogenetic protein 2 (BMP-2), a US Food and Drug Administration (FDA) approved drug, is a vital osteoinductive growth factor to treat bone defect via the recruiting of osteoblast progenitor cells. These cells induce angiogenesis and stimulate the osteogenic differentiation of MSCs^9,14,15^. However, the unwanted side effects of BMP-2, such as abnormal osteoclast differentiation, which results in increased bone resorption at the graft/implant site, can be expected on other cell types^16^.

Alendronate (ALN), which was approved by the FDA for clinical use in osteoporosis treatment, is a therapeutic agent used to treat osteoclast-induced bone loss by preventing the recruitment, differentiation, and bone-remodeling activity of osteoclasts, thereby expediting bone repair^17–19^. Additionally, according to Bone et al., ALN increases bone mineral density, which is especially effectual in the treatment of bone loss and osteoporosis^20^. The local and sustained delivery of ALN can be beneficial to the continuous inhibition of bone resorption during bone healing^21,22^.

Several groups have attempted to take advantage of the combination treatment of BMP-2 and ALN for bone regeneration^23,24^, disputing that the use of BMP-2 and ALN has no additive effects on bone regeneration for the following reasons: (i) ALN has a minimal effect on the differentiation and activation of osteoblasts, and (ii) the differentiation of osteoclasts and bone resorption by osteoclasts occurs after the intermediate stage of bone regeneration (~ 21 days). Therefore, the early release of ALN (i.e., the concomitant release of ALN) cannot suppress osteoclastic activity by excluding the optimal time of drug efficacy^25^. It has been suggested that this issue may be addressed by the encapsulation of ALN into microspheres prior to the combination with biodegradable scaffold^26^. Examples of such microspheres include poly(lactic-co-glycolic acid) (PLGA) microspheres, which have proven to be efficient in the delivery of biomolecules with controlled release for bone tissue engineering^27^.

**Figure 1:**
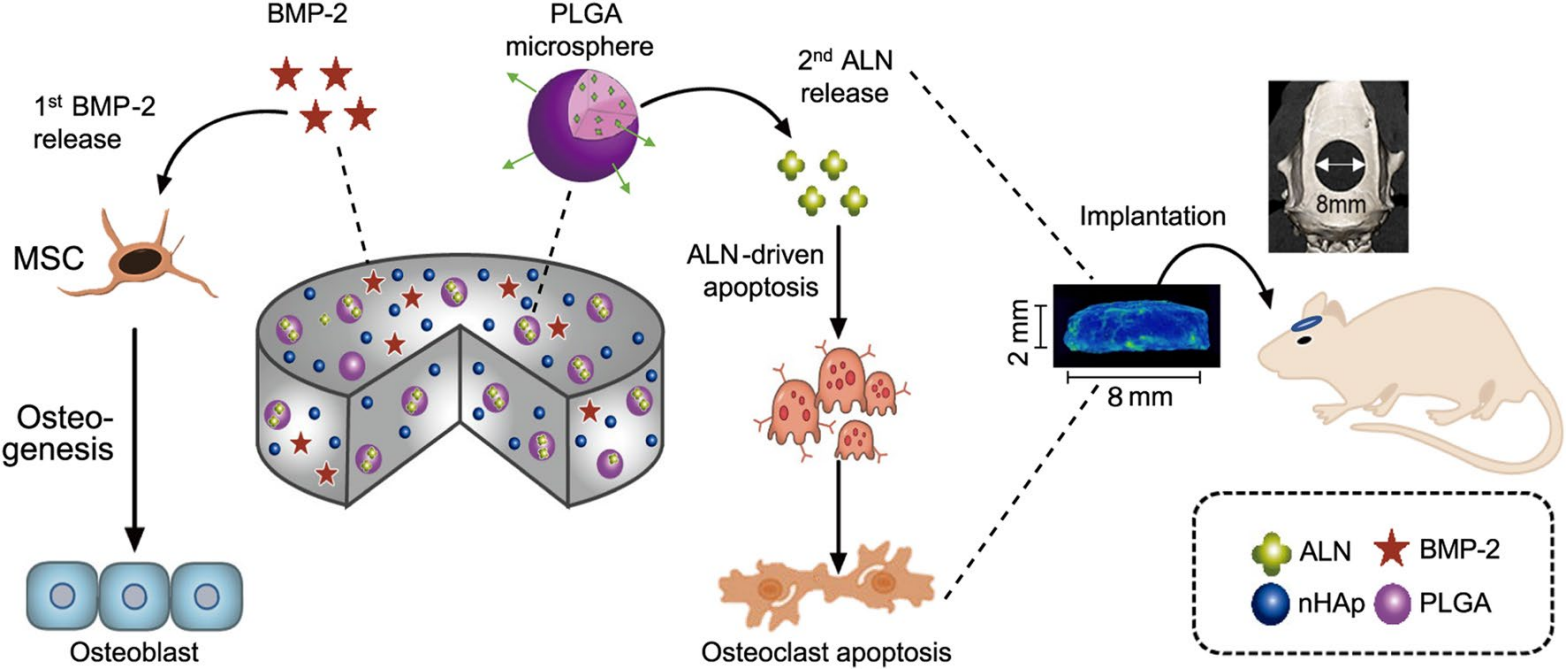
Schematic illustration of sequential dual-drug delivery using BMP-2 and ALN.

In this study, we developed a biodegradable sequential drug delivery scaffold for bone regeneration. A collagen-hydroxyapatite scaffold (CHAS) was used to incorporate PLGA microspheres encapsulating ALN, after which BMP-2 was loaded onto the scaffold. With the combination of BMP-2 and ALN in CHAS and sequential release, we expected a synergistic effect for bone regeneration. The first release of BMP-2 promotes the osteogenic differentiation of MSCs, and the secondary release of ALN inhibits osteoclastic activity, as shown in Fig. 1. The effects of the sequential release of BMP-2 and ALN on bone regeneration were evaluated in vitro and in vivo*,* compared to those of the single or concomitant release of BMP-2 and ALN using different and complementary assays, as shown in Table 1.

**Table 1:** Sample abbreviations used in this study.

Name of sample	Description of sample
Sham	Empty defect
Control	Original scaffold of CHAS without any drugs
ALN	Scaffold with physically adsorbed ALN
BMP-2	Scaffold with physically adsorbed BMP-2
B + A (P)	Scaffold with physically adsorbed BMP-2 and ALN
A + B (E)	Scaffold with physically adsorbed ALN and BMP-2 encapsulated in PLGA microspheres
B + A (E)	Scaffold with physically adsorbed BMP-2 and ALN encapsulated in PLGA microspheres

## Results

### Development of a sequential dual delivery system for BMP-2 and ALN in CHAS

In this study, we developed a CHAS-based sequential dual delivery system for BMP-2 and/or ALN using PLGA microspheres. First, ALN-loaded PLGA microspheres were fabricated using a W/O/W technique. Specifically, ALN was loaded into PLGA microspheres via the direct entrapment method, which enables its release in a sustained manner^28,29^. The diameter of the PLGA microspheres was controlled by adjusting the concentration of PLGA. During the fabrication of the PLGA microspheres, the ALN solution was mixed to prepare ALN encapsulated by the PLGA microspheres. Finally, the BMP-2 was physically adsorbed onto the CHAS, and the PLGA-encapsulating ALN was embedded onto the scaffold to obtain B + A (E). A CHAS without both BMP-2 and ALN was prepared as the control. In addition, we prepared the concomitant release of BMP-2 and ALN (B + A (P)), in which ALN was loaded onto the CHAS instead of being loaded into PLGA microspheres. The abbreviations associated with the experimental groups are indicated in Table 1.

**Figure 2:**
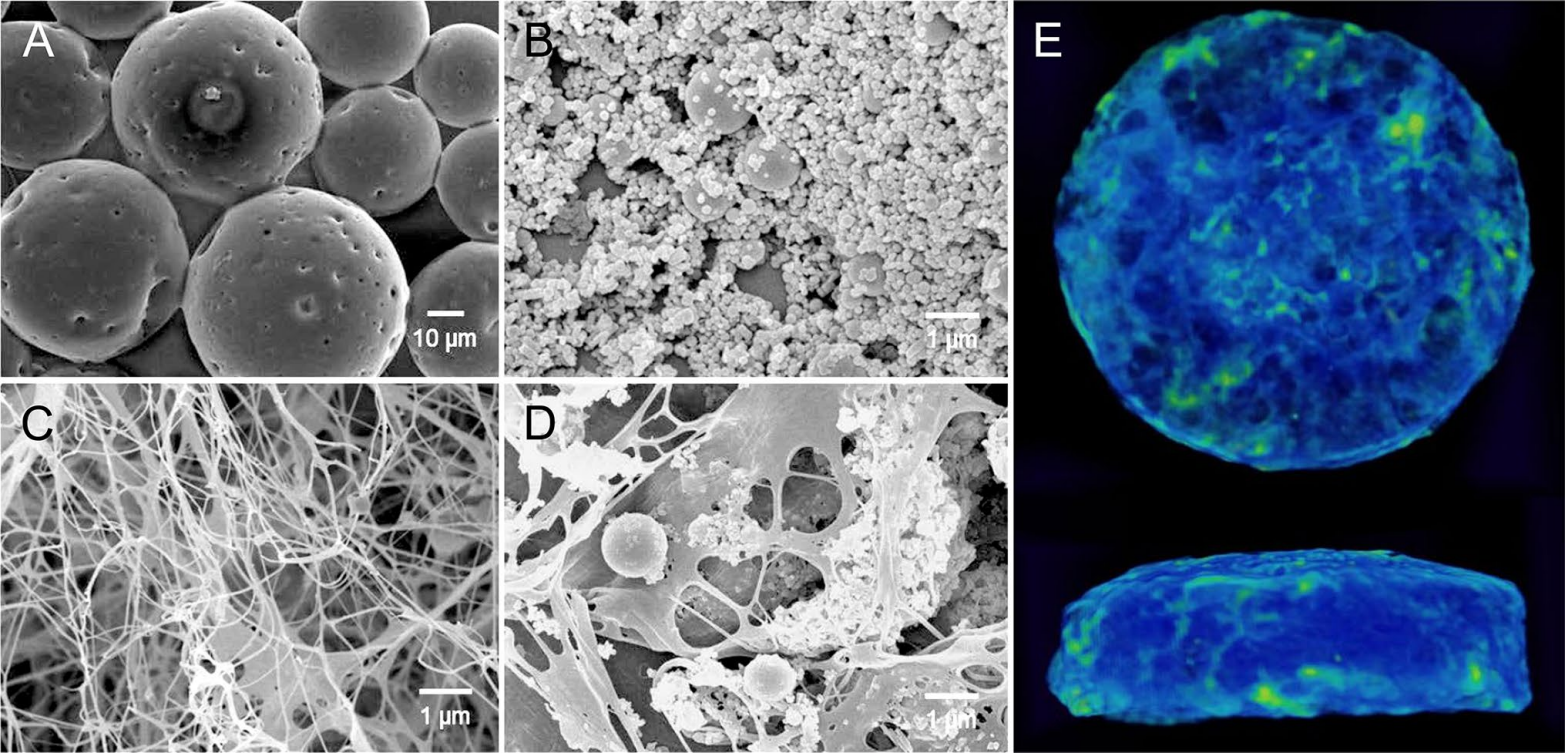
Morphological analysis of CHAS. SEM images of (**A**) PLGA microspheres; (**B**) hydroxyapatite; (**C**) collagen scaffold; (**D**) CHAS; (**E**) µ-CT images of CHAS (green threshold level at 147).

Next, we investigated the size and morphology of PLGA microspheres and nHAp, respectively (Fig. 2A, B). The PLGA microspheres had average diameters of 29.4 ± 8.64 μm. The morphology or size of the PLGA microspheres had no significant effect upon the loading of ALN or BMP-2 in the CHAS. Meanwhile, the pore size of the PLGA microspheres gradually increased with the degradation time (Supplementary Fig. S1). We used nano-sized nHAp (average diameter of 97.7 ± 57 nm) because a previous report demonstrated their improved osteoinduction of nano-sized nHAp compared to that of micro-sized HAp (Supplementary Fig. S2)^10^. The scanning electron microscopy (SEM) characterization of the CHAS showed that the microspheres and nHAps were homogeneously dispersed within the CHAS (Fig. 2C, D). As shown in Fig. 2E, the CT images show the side-view and top-view of the entire CHAS (8 mm (width) × 2 mm (height)).

### In vitro release of BMP-2 and ALN from CHAS

**Figure 3:**
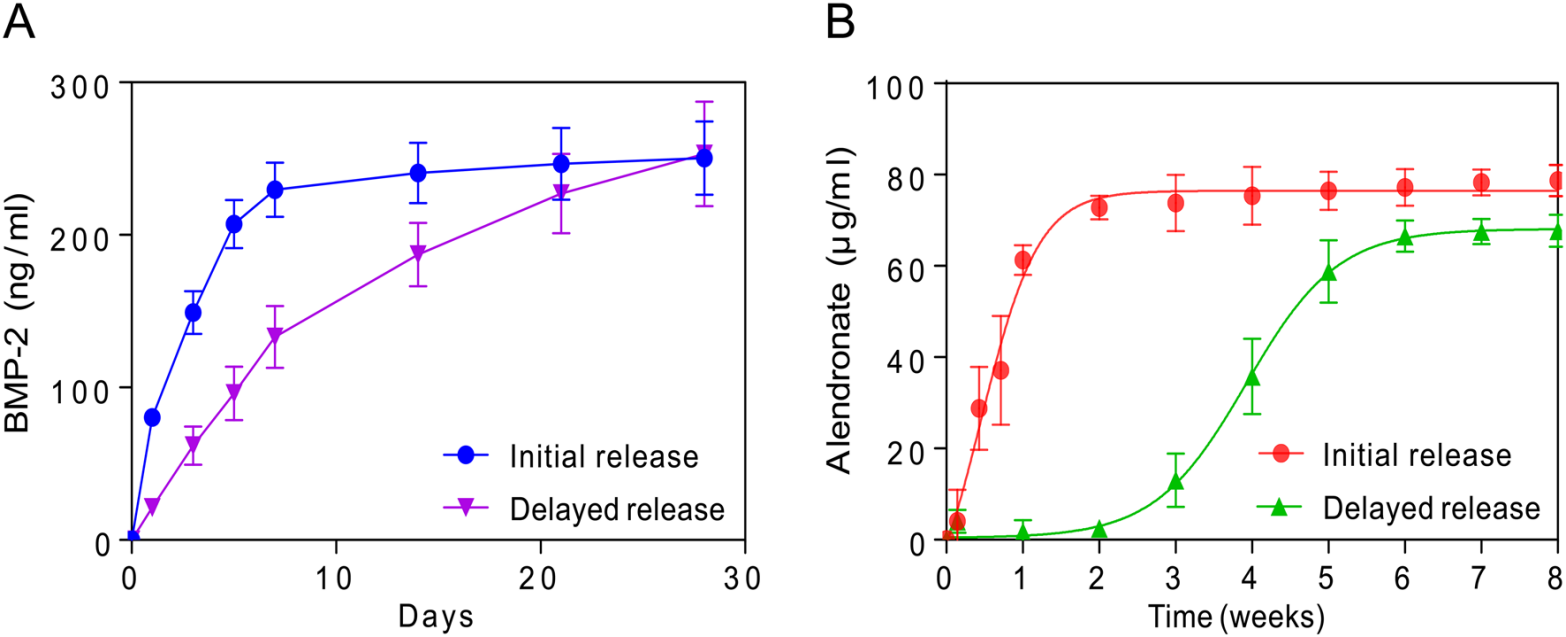
Initial or delayed drug release of BMP-2 (**A**) and alendronate (**B**) depending on the presence of PLGA microparticles in CHAS.

The release profiles of BMP-2 and ALN from the CHAS loaded in vitro were analyzed using BMP-2 ELISA kits and the spectrophotometric method, respectively (Fig. 3). A rapid release with 30% of the total BMP-2 from the scaffold was observed during the first day, followed by ~ 90% of the cumulative release after 7 days. In contrast, the release of BMP-2 (E) encapsulated in PLGA microspheres from the scaffold was sustained compared to that of BMP-2. A lower initial burst release with 10% of the total amount of BMP-2 (E) was identified on the first day, and BMP-2 was released for up to four weeks. At 1 week, almost 90% of BMP-2 without PLGA microspheres was released from the scaffold, whereas the remaining percentage of BMP-2 with PLGA microspheres was considerably higher than 50%. This result indicates that the release was delayed by the PLGA microspheres. Similarly, distinct release profiles of ALN within or outside of PLGA microspheres from the scaffold were observed (Fig. 3B). A burst release of ALN (60%) from the scaffold was observed on the first day. The cumulative release of ALN was saturated when 90% of the total ALN was released after 2 weeks. In contrast, the ALN encapsulated in PLGA microspheres showed relatively slow and sustained release compared to the ALN outside PLGA microspheres^30^. Initially, ALN within PLGA microspheres was rarely released from the scaffold for 2 weeks. After 2 weeks, ALN was released subsequently for 8 weeks. These results indicate that the release of ALN and BMP-2 from the scaffold can be significantly delayed because of the encapsulation of drugs in PLGA microspheres^31^.

### In vitro osteogenesis study

**Figure 4:**
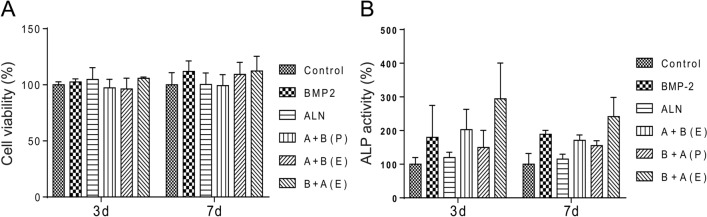
(**A**) Cell viability evaluation of CHAS with drugs (i.e., BMP-2 and/or ALN) using CCK-8 assay on osteoblasts. (**B**) ALP activity of osteoblasts in CHAS depending on the drugs (i.e., BMP-2 and/or ALN).

The proliferation of pre-osteoblasts on the scaffold loaded with BMP-2 and/or ALN was assessed using a CCK-8 assay from 3 and 7 days. In Fig. 4A, the pre-osteoblast cells exhibit adequate proliferation ability on scaffolds loaded with BMP-2 and/or ALN for both 3 and 7 days. The results indicate that the CHAS with both bioactive molecules (i.e., BMP-2 and ALN) has favorable biocompatibility. The osteogenic property of scaffolds was also analyzed, and the ALP activity of the pre-osteoblast culture with scaffolds with BMP-2 and/or ALN was studied (Fig. 4B). It was seen that all scaffolds containing BMP-2 showed higher ALP activity than scaffolds without BMP-2. The scaffolds of B + A (E) exhibited the highest level of ALP activity among all groups.

### µ-CT analysis

**Figure 5:**
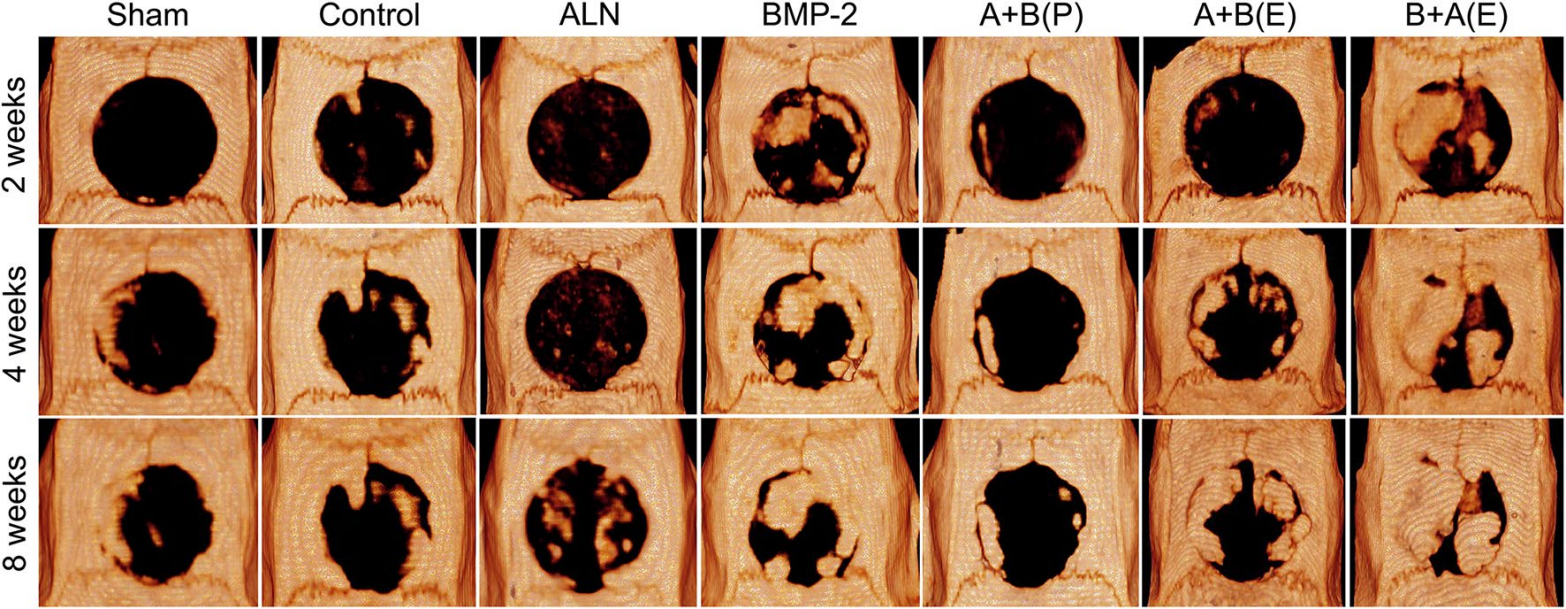
Micro-computed tomography-reconstructed images at 2, 4, and 8 weeks postoperatively. µ-CT radiographic images of rat calvarial bone formed at 2, 4, and 8 weeks postoperatively in different groups, showing degree and extent of mineral deposition at the defect site.

**Figure 6:**
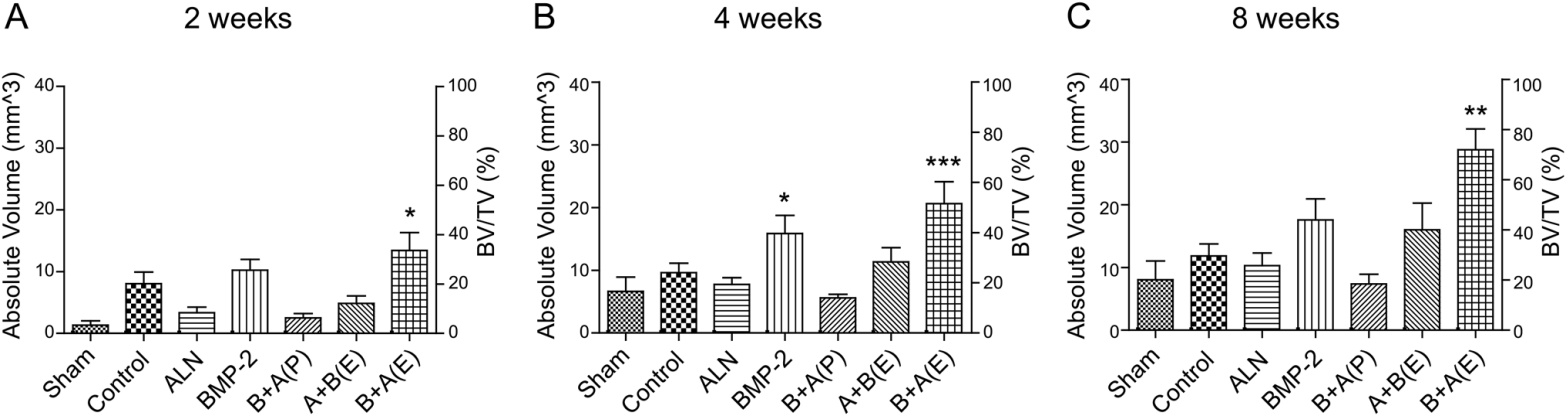
Quantitative analysis of new bone volume at 2, 4 and 8 weeks postoperatively. µ-CT based histomorphometry analysis of the defect site showing the amount of highly mineralized volume and BV/TV percentage at the defect site at each time point (2, 4, and 8 weeks). (*p < 0.05; **p < 0.01; ***p < 0.001 vs. control group; one-way ANOVA test).

We evaluated the accumulation and maturation of bone-like tissue in the calvarial bone defect using in vivo µ-CT imaging at 2, 4, and 8 weeks after implantation. Figure 5 shows that the bone content increased in the scaffolds of BMP-2 and B + A (E) compared to the other scaffolds at 2, 4, and 8 weeks. Additionally, new bone was generated from the edge of the defect to the center. The bone volume fraction was the highest in the scaffold of B + A (E), where BMP-2 and ALN were sequentially released (Fig. 6). We analyzed the bone volume/total volume (BV/TV) increment of BMP-2 and the B + A (E) scaffold compared to the control group. The BV/TV increments of BMP-2 and the B + A (E) scaffold were 27.16% and 35.62% (*p* < 0.05), respectively, in 2 weeks. That of BMP-2 and the B + A (E) scaffold were 41.99% and 54.71% (*p* < 0.01), respectively, in 4 weeks. Finally, that of BMP-2 and the B + A (E) scaffold were 46.58% and 76.23% (*p* < 0.001), respectively, in 8 weeks. In contrast, the increase in bone content of the BMP-2 scaffold was negligible at 8 weeks (46.58%) compared to the significant increase in 4 weeks of 41.99% (*p* < 0.05) (Fig. 6B, C). Considering the saturation of BMP-2 release after 2 weeks, as shown in Fig. 3A, these results support the hypothesis that the delayed release of ALN-encapsulated PLGA microspheres further enhances bone regeneration at the graft site. Notably, the average BV/TV of the B + A (E) group was statistically higher than that of all other animal groups (Fig. 6A–C).

### Histological analysis

**Figure 7:**
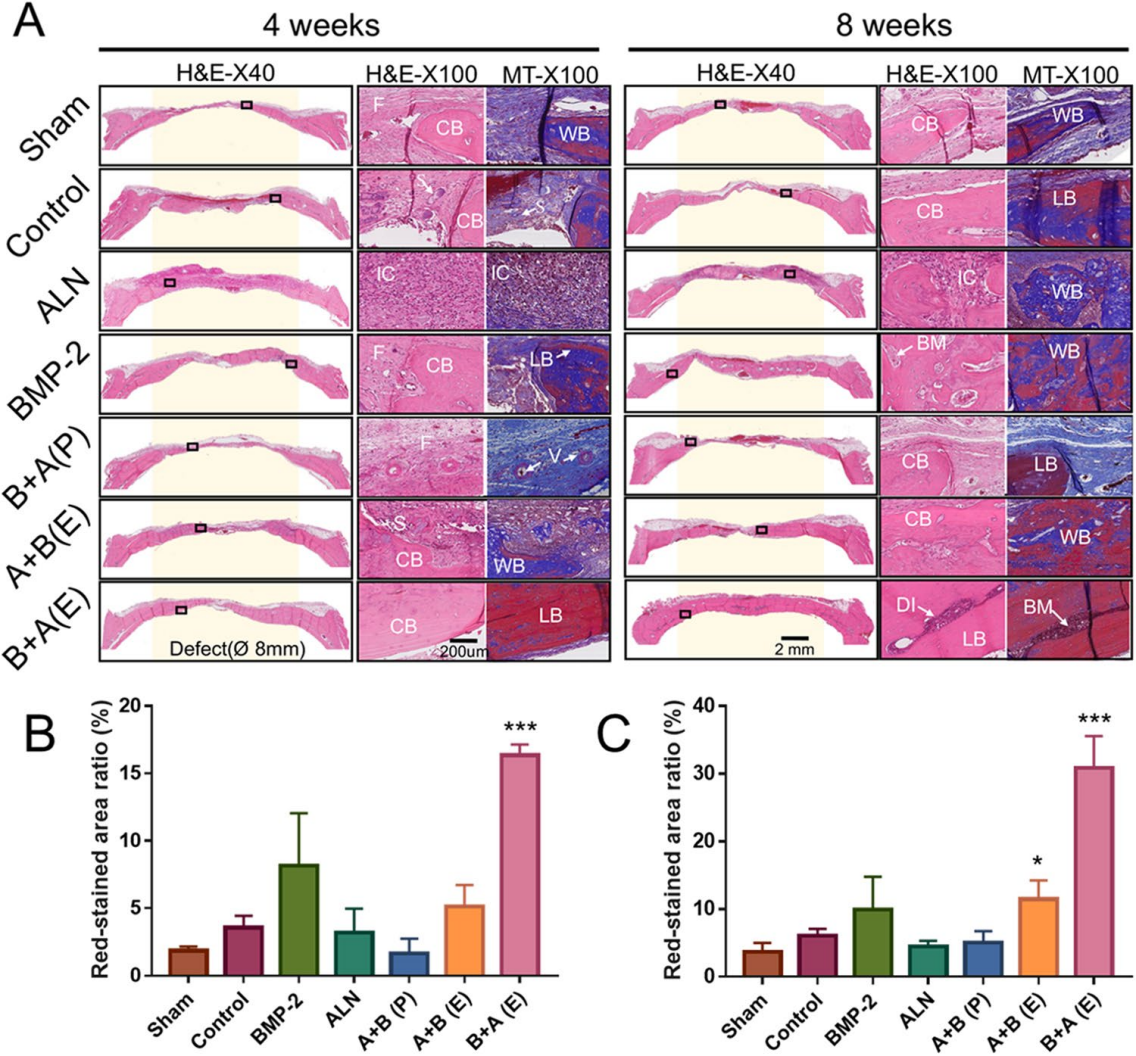
Histological evaluation of bone regeneration in rat calvarial defects at 4 and 8 weeks postoperatively: (**A**) Image for hematoxylin and eosin staining and Masson’s trichrome staining. Yellow background shows where defects were created. Semi-quantitative analysis of red-stained area ratio (%) of Masson’s trichrome staining in (**B**) 4 weeks and (**C**) 8 weeks using ImageJ software (V1.8.0_172, NIH, USA) (https://imagej.nih.gov/ij/). CB, cortical bone; LB, lamellar bone; WB, woven bone; F, fibrous tissue; IC, inflammation cell; S, scaffold; BM, bone marrow; DI, diploe; V: vessels. (×40 and ×100 magnification, scale bar = 200 μm. *p < 0.05; ***p < 0.001 versus control group; Two-way krANOVA test).

To determine whether a sequential release of BMP-2 and ALN induces mature bone-like tissue on the calvarial defect site, histological examinations were performed. At 4 and 8 weeks after surgery, several defect sites in the sham, control, and ALN groups were filled with fibrous connective tissue without much osseous tissue (Fig. 7A). Notably, the ALN, A + B (P), and A + B (E) groups showed several inflammatory cells along with fibroblast-like cells inside the defect area, preventing the formation of new bone. The results indicate that the burst release of ALN without PLGA microspheres induces an inflammatory response on the graft site as a side effect of bone regeneration. Furthermore, no indication of heterotopic ossification outside of the defect area was observed. In compliance with our µ-CT measurement, the B + A (E) group exhibited considerable amount of mature bone with lamellar structure hematoxylin and eosin (H&E) and Masson’s trichrome (MT) staining compared to the other groups at 8 weeks. Moreover, the defects were almost covered with compact bone, including the diploe, which resembles the natural structure of the original skull. In Fig. 7B, C, the red-stained area ratio of the MT staining demonstrated a significant increase at both 4 and 8 weeks (*p* < 0.001), indicating that the degree of maturity increased with time.

## Discussion

Engineered biomaterials for in situ tissue engineering are a powerful tool for treating bone defects by ready-to-use biodegradable, cell-free constructs that are designed to promote bone regeneration with bioactive molecules (e.g., growth factors). The goal of this work was to develop a cell-free composite scaffold to initially stimulate osteoblastic activity and subsequently inhibit osteoclastic activity via ALN-driven osteoclastic apoptosis. To accomplish this design, a CHAS was used to incorporate ALN-loaded PLGA microspheres and BMP-2 to achieve sequential drug delivery scaffolds (B + A (E)) for enhanced bone regeneration used in in situ tissue engineering.

It was observed that the drug release profiles of BMP-2 and ALN had different patterns. Only ALN achieved a distinct sequential release because ALN is a small molecule (molecular weight: 249.1 g/mol). It has been reported that ALN with PLGA microspheres was released in a prolonged and sustained manner^32^. On the other hand, BMP-2 is a protein with a molecular weight in the range of 30–38 kDa. Furthermore, it has been reported that BMP-2 with PLGA microspheres was released in a few days^33^. As a result, the drug release profiles differ according to the types and characteristics of the drugs.

For comparison, we prepared the B + A (P) scaffold in which ALN and BMP-2 were concomitantly released from CHAS. In this scaffold, ALN was physically adsorbed onto the scaffold instead of encapsulating the PLGA microspheres onto the composite scaffold. The BMP-2 encapsulation efficiency of PLGA microspheres prepared by the W/O/W technique was considered to be 25%. In contrast, the ALN encapsulation efficiency of PLGA microspheres prepared by the W/O/W technique was 78%. These results indicate that PLGA microspheres have a high loading efficiency for ALN.

To analyze the effects of each drug on the cell viability and osteogenic activity of osteoclasts in vitro, the proliferation and ALP activity of osteoprogenitor cells seeded in each scaffold were measured. The scaffolds containing BMP-2 showed higher osteoblastic activity than that of CHAS with ALN, which implied that ALN alone has a limited effect on osteoblastic differentiation and activation in vitro. This could be due to the direct effects of ALN on the differentiation and activation of osteoclasts, but not on osteoblasts^34,35^.

To evaluate the synergistic effect of bone regeneration using BMP-2 + ALN, each scaffold was implanted with BMP-2 and/or ALN in a rat cranial defect model. At 8 weeks after the implantation of the B + A (E) scaffold. The extent of new bone formation was in the following order: B + A (E) > BMP-2 > A + B (E) > Control > ALN > Sham > B + A (P). It is speculated that the burst release of ALN from CHAS hinders bone regeneration. In addition, excessive inflammation was observed, which is an interleukin-1-dependent mechanism^36^ by the uncontrolled and large amount of nitrogen-containing ALN. Accordingly, the local and delayed release of ALN plays a pivotal role in eliciting its positive effect (i.e., inhibition of osteoclastic activity on bone regeneration). Moreover, considering that BMP-2 causes a side effect in the osteoclast differentiation, ALN provides synergistic effects by inhibiting osteoclastic differentiation induced by BMP-2. Taken together, the sequential release of BMP-2 and ALN (B + A (E)) demonstrates a synergistic effect on bone regeneration without an excessive inflammation response and fibrous barrier at the graft site (Fig. 7). In general, flat cranial bones occur in intramembranous bone formation. With a focus on craniofacial plastic surgery, the CHAS scaffold was created as a carrier specialized for the intramembrane bone formation system. In this respect, the experimental setup has limitations in terms of its application to other bone defects or fractures.

Angiogenesis and vascularization capability are important for the practical application of engineered grafts by enhancing bone fracture repair and regeneration^37^. Histological analysis (Fig. 7) shows the presence of blood vessels and bone marrow in newly formed bones. Moreover, in the case of the B + A (E) group, the defected area recovered into a spongy bone, which was covered on either side by a layer of compact bone resembling the structural characteristics of natural skull bones. Such a composite system demonstrates the potential for bone regeneration applications without any side effects resulting from BMP-2.

## Conclusion

We developed a collagen-hydroxyapatite scaffold incorporated with BMP-2 and ALN-loaded PLGA microspheres for the sequential release of drugs to control bone remodeling. BMP-2 promotes osteogenesis by activating osteoblast and ALN inhibits osteoclast-mediated bone resorption. Subsequently, we demonstrated that our system (B + A(E)) accelerates bone formation more than the single-use of BMP-2, a conventional treatment. Our results indicate the improved functionality and safety of a bio-inspired sequential release system using BMP-2 and ALN, with a synergistic effect on bone regeneration both in vitro and in vivo. Although the mechanical properties of CHAS were improved by the addition of nHAp, these properties still need to be increased when it is being applied to a load-bearing area. Additionally, we established the biocompatibility of the biomaterial as well as a positive bone healing effect. The composite scaffold with sequential dual-drugs delivery is a promising platform for bone regeneration.

## Materials and methods

### Materials

We purchased rat tail-derived collagen type I solution (3.8 mg/ml) and neutralization solutions from Advanced BioMatrix (CA, USA) for 3D structured hydrogels. We purchased recombinant human bone morphogenetic protein-2 (rhBMP-2) from Peprotech (NJ, USA). We purchased nano-hydroxyapatite particles (nHAp) from Sigma-Aldrich, USA, and confirmed that their sizes were within ~ 200 nm using SEM (Supplementary Fig. S1). The cell counting kit-8 (CCK-8) and the rhBMP-2 ELISA kit were purchased from Goma Biotech, Korea. Carboxyl-terminated 50:50 PLGA (0.67 dl/g) was purchased from LACTEL Absorbable Polymers. Polyvinyl alcohol (M_w_ = 13,000–23,000), dichloromethane (DCM), ALN, sodium hydroxide, 2-mercaptoethanol (2ME), and ortho-phthalaldehyde (OPA) were purchased from Sigma-Aldrich, Korea. The Dulbecco's modified Eagle's medium (DMEM), fetal bovine serum (FBS), and penicillin–streptomycin were purchased from Gibco.

### CHAS fabrication

A CHAS was fabricated with the omission of the chemical crosslinking step to avoid the protein loss in the solution prior to its use in vitro and in vivo. Then, 2 mL of collagen solution (3.8 mg/mL) was added to each vial and the collagen solution stirred with 100 mg of nHAp nanoparticles (< 200 nm). The entire fabrication process was conducted at a temperature of 4 ˚C to avoid the unintended gelation and heat denaturation of collagen. This suspension was blended at 15,000 rpm for 20 min using an overhead blender in a reaction vessel that was kept at 4 °C to prevent heat denaturation of the collagen. The slurry was degassed in a vacuum to remove air bubbles, which may result in uncontrolled porosity. To incorporate rhBMP-2 into the scaffold, rhBMP-2 (R&D Systems, UK) and PLGA microparticles were gently mixed with the CHAS slurry before the degassing step at a final dose of 1 μg rhBMP–2/ml slurry, and 150 μL of the slurry was used to produce a scaffold in an 8 × 2 mm polydimethylsiloxane (PDMS) mold (1 μg per scaffold). The dose of BMP-2 was chosen via in vitro cell viability and ALP activity assay of osteoblast using a microscope (Supplementary Fig. S5). The in vitro BMP-2 and ALN release of a series of CHAS were analyzed prior to this study. After gelation for 3 h, the CHAS was lyophilized at − 80 °C using a constant cooling freeze-drying protocol. Subsequently, the scaffolds were sterilized using a UV light for 4 h (λ = 254 nm) at 4 °C^38^.

### Drug release kinetics of BMP-2 in CHAS

To examine the BMP-2 release profile of CHAS, the hydrogels were placed in 3 mL of phosphate buffered saline (PBS) and into a shaking incubator (WIS-20, Daihan Scientific, Korea) at 37 °C at 120 rpm. At given time points, up to 28 days, release media (i.e., PBS buffer) were extracted and replaced with fresh PBS buffer. The protein concentration released from the CHAS was determined using an rhBMP-2 ELISA kit (KOMA Biotech, Korea) according to the manufacturer’s instructions via a microplate reader (Versa Max, Molecular Devices, CA, USA).

### Drug release kinetics of ALN in CHAS

The spectrophotometric method was used to quantify the release of ALN gradually. Here, a working solution was freshly prepared by dissolving 10 mg of anhydrous OPA in 10 mL of 0.05 M NaOH, after which 50 µL of 2ME solution was added. Standard solutions of ALN were prepared by dissolving ALN, after which they were diluted. For the calibration curves, standard solutions containing ALN and the working solution were mixed in a 1:1 volume ratio and incubated for 60 min at 37 °C. The resulting solutions were registered at 335 nm using a multi-plate reader. ALN released from the CHAS was measured through the same procedure and quantified using the calibration curves.

### Preparation of PLGA microspheres and embedding to CHAS

PLGA microspheres containing BMP-2 or ALN were prepared using a water-in-oil-in-water (W/O/W) technique. PLGA polymer (400 mg) was dissolved in DCM (10 mL). ALN solubilized in 0.05 M NaOH (0.25 mL, 4% w/v) or BMP-2 solubilized in PBS was added to the polymer solution and homogenized for 1 min at 3000 rpm to form a primary emulsion (W/O). The polymer solution in DCM was added dropwise to 4 mL of a 0.5% aqueous solution of polyvinyl alcohol (PVA). The mixture was then stirred in open air for 6 h at 600 rpm using a magnetic stirrer to evaporate the organic solvent. The pore size of PLGA microspheres, depending on the degradation time, was characterized using a scanning electron microscope (Supplementary Fig. S2).

### Cell cultures and cell viability test

The osteoblasts (MC3T3-E1) and osteoclast precursors (RAW 264.7) were maintained in the DMEM supplemented with 20% FBS and 100 units/mL of penicillin–streptomycin with 5% CO_2_ at 37 °C. Cells were passaged at 70 − 90% confluency. A CCK-8 cell viability kit was employed to determine cell viability. Cells with a density of 2.0 × 10^4^ cells/well were incubated on the CHAS for 3 and 7 days, respectively, for viability tests. CCK-8 reagent (10 µL) was added to the cells suspended in serum-free media and incubated for 3 h at 37 °C. The CCK-8 reagent (WST-8) was reduced by dehydrogenases in a cell formed formazan that was yellow. The intensity of the resulting color was read at an absorbance of 450 nm with a reference wavelength of 650 nm using a microplate reader (Molecular Devices, USA).

### Alkaline phosphatase (ALP) activity assay

To measure the ALP activity of osteoblasts, frozen pnitrophenyl phosphate (pNPP) was freshly equilibrated at room temperature. The cells seeded on each CHAS for 3 and 7 days, respectively, were lysed using a cell lysis buffer with sonication. pNPP was added to the cell lysed buffer and incubated in the dark for 30 min at room temperature. The intensity of the resulting color was registered at 405 nm using a microplate reader.

### Animal model and surgical procedures

Sprague–Dawley rats (Forty-two males, 9–10 weeks old, weighing 300–350 g, (Koatech, Keung-Ki, Pyong-Taek, Korea)) were used in this study. All animal experiments were approved by the Institutional Animal Care and Use Committee (IACUC No. 35-2019-0072) at Seoul National University Hospital. All experiments were performed in accordance with relevant guidelines and regulations, including ARRIVE. The animals were provided with food and water ad libitum. Three batches were tested for each sample type. We initially created a critically sized calvarial bone defect in rats, as previously reported by Spicer et al. and Kim et al.^39,40^. Rat anesthesia was induced using isoflurane, after which alfaxalone (ALFAXAN CIV 80 mg/kg) and xylazine (Rompun, 10 mg/kg) were injected intraperitoneally for deep anesthesia. After anesthesia, the hair on the scalp covering the calvarial vault was shaved, followed by cleaning using Betadine (Hyundai Pharm, Korea).

A subcutaneous injection containing 0.3 ml of 2% lidocaine with 1:100,000 epinephrine was administered as a local anesthetic along the sagittal midline of the skull. A sagittal incision of 4 cm in length was made over the scalp from the nasal bone to the middle sagittal crest. Subsequently, a full-thickness flap was elevated, and the periosteum was dissected to expose the cranial bone (Supplementary Fig. 3A). An electrical bone trephine bur (Seoul, South Korea) was used to create an 8 mm critical-sized defect under sterile saline irrigation (Supplementary Fig. 3B, C). The calvarium was excised and extensive care was taken to avoid damage to the dura mater (Supplementary Fig. 3D, E). The animals were randomly divided into seven groups (n = 6 per group); (π) Sham/empty defect, (θ) Control/vehicle control, (ρ) ALN, (σ) BMP-2, (τ) A + B (P), (υ) A + B (E), and (vii) B + A (E) (Table 1). Next, the samples were randomly assigned to each group (Supplementary Fig. 3F). After implanting the surrounding soft tissue and suturing the skin, the animals were provided with analgesics until recovery, (Supplementary Fig. 3G, H). There were no adverse events in the post-operation.

### Micro-computed tomography (µ-CT) analysis

To evaluate new bone formation, images from the bone defect were obtained using micro-computed tomography (µ-CT; NFR Polaris-G90, Nano Focus Ray, Korea) at 2, 4, and 8-weeks post-operation. Imaging was performed on the scanner at an isotropic voxel size of 9 μm with an X-ray tube current of 180 μA and a voltage of 55 kV. At each time point for µ-CT imaging, the animals were anesthetized using the aforementioned protocol. New bone formation in the defects was evaluated using the AMIRA software (Version 5.4, ZIB & Visage Imaging, Germany). To measure newly formed bone, a circular area of predefined size was selected as the region of interest (ROI) in the two-dimensional images.

The pixel zone representing ossification in the defined ROI was then reconstructed in 3D by creating a volume of interest (VOI) in the lower and upper ranges of the threshold using grayscale units, where the new bone formation was determined by setting the gray threshold level at 1000. The new bone volume fracture (BV/TV) percentage was calculated by dividing the newly formed BV by that of the original TV. (Supplementary Fig. 4).

### Sample preparation and histological analysis

To confirm further the formation of new bone, histological evaluations using hematoxylin and eosin (H&E) and Masson’s trichrome (MT) staining were performed at 4 and 8-weeks post-injection. The animals were euthanized using CO_2_, and the defect sites were removed along with the surrounding bone and soft tissues. The harvested specimens were soaked in 10% (v/v) buffered neutral formalin (Sigma-Aldrich, USA) for 3 days, decalcified for 7 days, sectioned into 5 μm thick sections, and stained through H&E or MT staining. MT staining discriminates between non-mineralized osteoid and connective tissue, which are highlighted in blue, and the mineralized bone, which is highlighted in red. The semi-quantitative detection of the red-stained area ratio (%) of MT staining was performed using ImageJ software (V1.8.0_172, NIH, USA). For histologic evaluation, the slides were examined by a professional pathologist using an optical microscope (BX53F, OLYMPUS, Japan) with × 40 and 200× magnification levels.

### Statistical analysis

The experiments were performed thrice. Statistical analysis was performed using prism7 software (GraphPad Software, Inc., USA) to evaluate the differences between the groups using analysis of variance (ANOVA). Differences at *p < 0.05, **p < 0.01, and ***p < 0.001 were considered statistically significant. The size of nHAps and the pore distribution of PLGA microspheres and MT stained area were analyzed by ImageJ software (V1.8.0_172, NIH, USA).

## Supplementary Information

Supplementary Figures.
