# Supplementary material for: Sequential dual-drug delivery of BMP-2 and alendronate from hydroxyapatite-collagen scaffolds for enhanced bone regeneration
Source: Sci Rep. 2021 Jan 12;11:746. doi: 10.1038/s41598-020-80608-3 (PMC7804460; doi:10.1038/s41598-020-80608-3)
Supplement: Supplementary file 1 — Supplementary Figures. [file 41598_2020_80608_MOESM1_ESM.docx]

**Supplementary information**

**for**

**Sequential** **dual-drug delivery of BMP-2 and alendronate from hydroxyapatite-collagen scaffolds for enhanced bone regeneration**

Dongtak Lee^a, ‡^, Maierdanjiang Wufuer^b, ‡^ , Insu Kim^a, ‡^, Tae Hyun Choi^b^, Byung Jun Kim^b^, Hyo Gi Jung^a^, Byoungjun Jeon^c^ , Gyudo Lee^d^, Ok Hee Jeon^e^, Hak Chang^2,*^ and Daesung Yoon^a,f,*^

^a^ School of Biomedical Engineering, Korea University, Seoul 02841, Republic of Korea

^b^ Department of Plastic and Reconstructive Surgery, College of Medicine, Seoul National University, Seoul, 110-799, Republic of Korea

^c^ Interdisciplinary Program in Bioengineering, Graduate School, Seoul National University, Seoul, 110-799, Republic of Korea

^d^ Department of Biotechnology and Bioinformatics, Korea University, Sejong 30019, Republic of Korea

^e^ Department of Biomedical Sciences, College of Medicine, Korea University, Seoul 02841, Republic of Korea

^f^ Interdisciplinary program in Precision Public Health, Korea University, Seoul 02841, Republic of Korea

^‡^These authors contributed equally to this study.

^*^Corresponding authors: D. S. Y. ([dsyoon@korea.ac.kr](mailto:dsyoon@korea.ac.kr)); H. C. (hchang@snu.ac.kr)


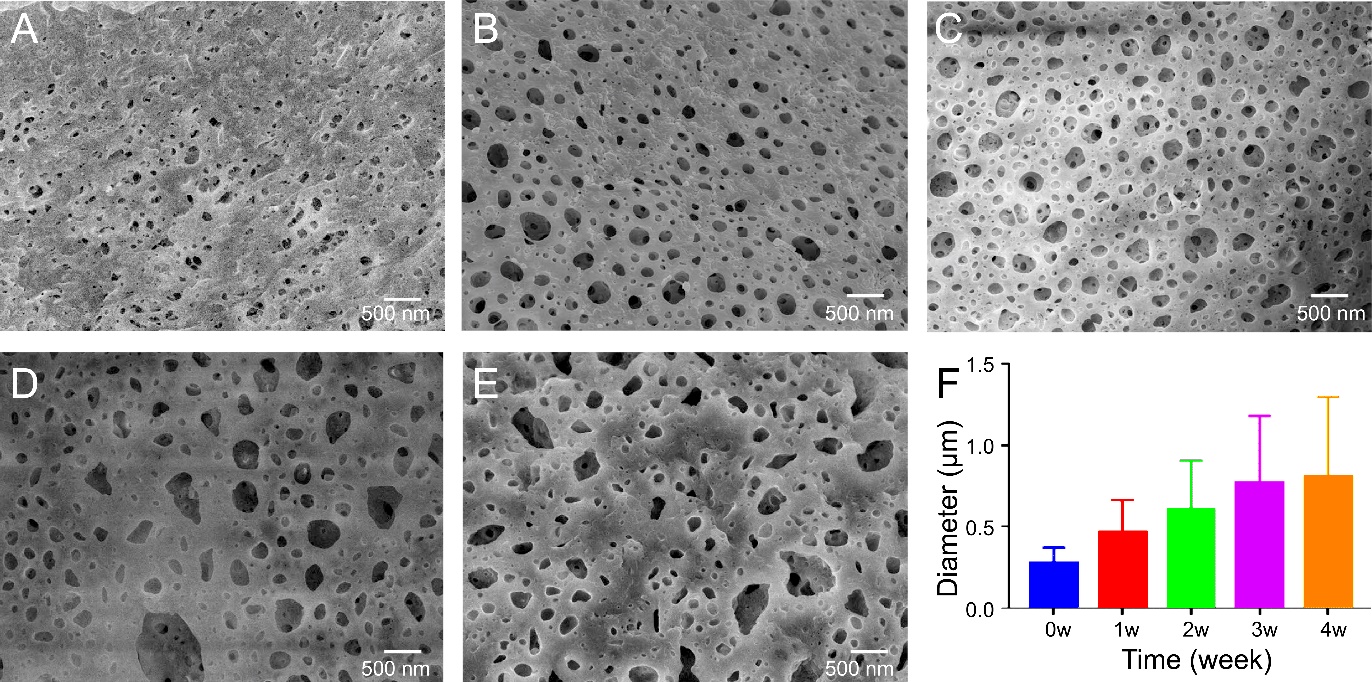


Supplementary Figure S1. SEM image and pore size distribution of PLGA microspheres. SEM images show the pore size of PLGA microspheres after (A) 7 days, (B) 14 days, (C) 21 days, (D) 28 days, and (E) 35 days. (F) The pore size distribution of PLGA microspheres obtained from the SEM images. The pore size of PLGA are using ImageJ software (V1.8.0_172, NIH, USA) (<https://imagej.nih.gov/ij/>).

.


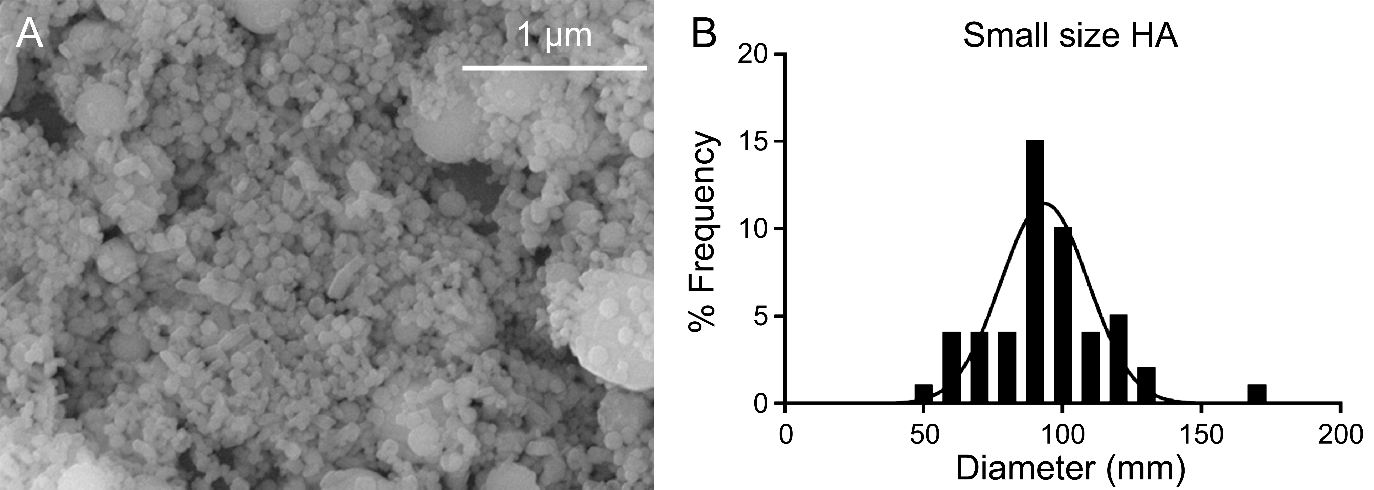


Supplementary Figure S2. SEM image and size distribution of hydroxyapatite nanoparticles (nHAps). (A) SEM image shows the size of nHAps adopted to fabricate CHAS. (B) The size distribution of nHAps obtained from the SEM image was fitted to a Gaussian function. The diameter of nHAps are using ImageJ software (V1.8.0_172, NIH, USA) (<https://imagej.nih.gov/ij/>).

.


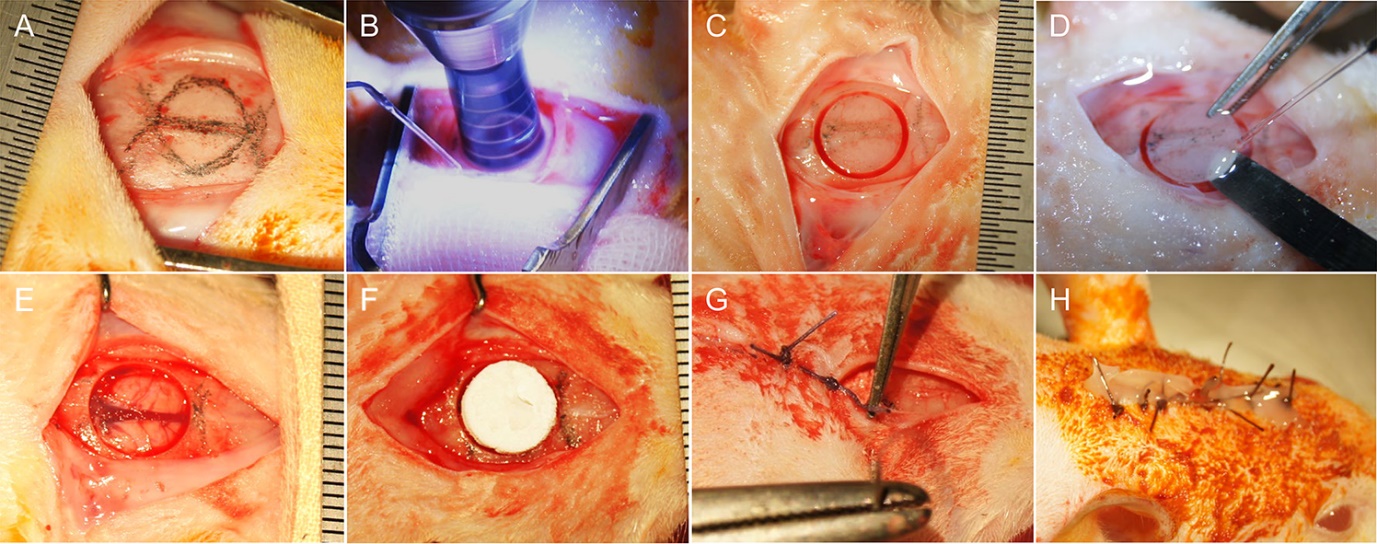


Supplementary Figure S3. Surgical procedure for creating an 8 mm cranial defect in rat and implantation of the scaffold at the calvarial defect site. A sagittal incision of 4 cm in length was made over the scalp from the nasal bone to the middle sagittal crest, and a full-thickness flap was elevated and the periosteum was dissected to expose the cranial bone (A). An electrical bone trephine bur was used to create an 8 mm critically sized calvarial defect under sterile saline irrigation (B, C). The calvarium was excised and great care was taken to avoid damage to the dura mater (D, E). Then, the scaffolds were randomly implanted into the defect (F). After implanting the surrounding soft tissue and suturing the skin, the animals were provided with analgesics until recovery (G, H).


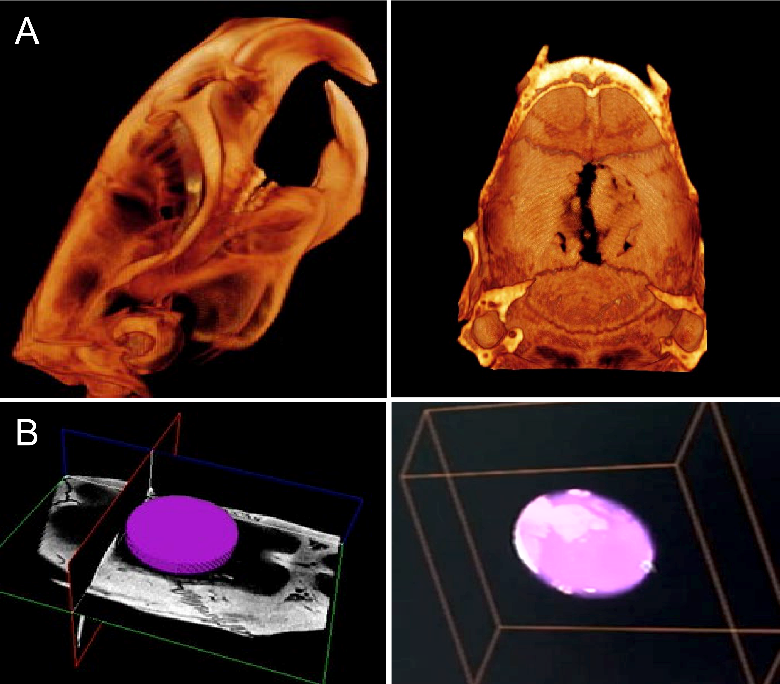


Supplementary Fig S4. Micro-CT imaging procedures to detect bone regeneration. (A) Three-dimensional tomography radiological reconstructive bone imaging was used for bone surface regeneration measurements. (B) 3D-selected circular defect area. New bone formation in the defects was evaluated using the AMIRA software (Version 5.4, ZIB & Visage Imaging, Germany).


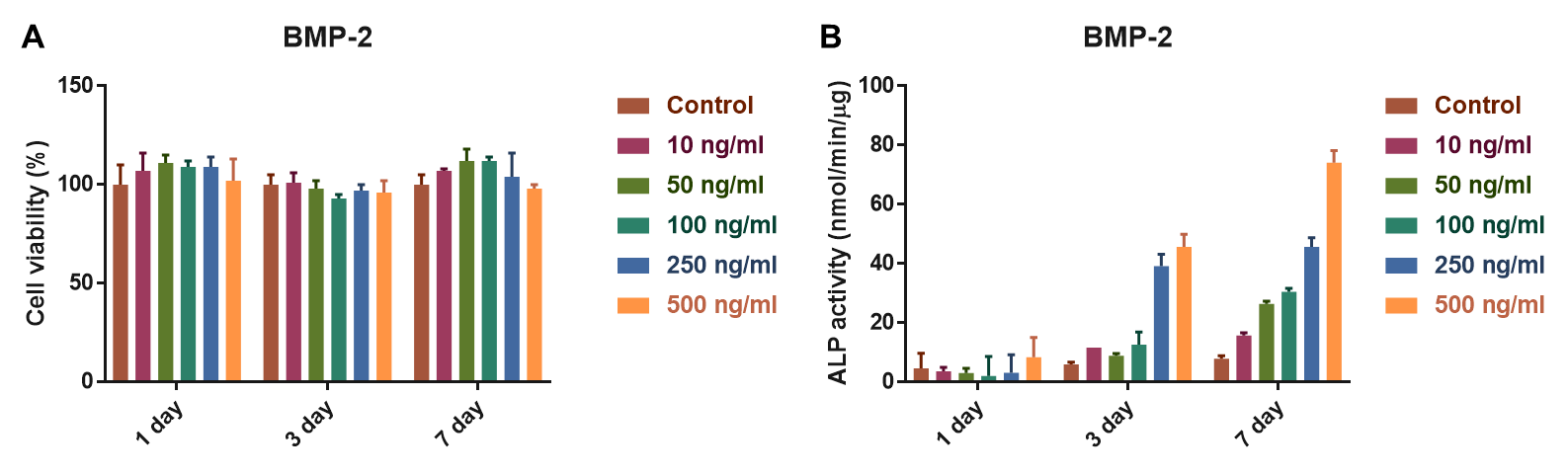


Supplementary Fig S5. Cell viability and ALP assay of osteoblast suspended in various concentration of BMP-2.
